# Secondary Malignant Transformation of Giant Cell Tumor of Bone Nine Years After Initial Curettage: A Case Report and Literature Review

**DOI:** 10.3390/reports9030202

**Published:** 2026-06-25

**Authors:** Ibrahim S. Alshaygy, Mishari N. Alanezi, Omar A. Aldosari, Safana M. Alomar, Hatim A. Khoja

**Affiliations:** 1 Department of Orthopedic Surgery, College of Medicine, King Saud University, Riyadh 11362, Saudi Arabia; 2 College of Medicine, King Saud University, Riyadh 11362, Saudi Arabia; 3 Department of Pathology, King Faisal Specialist Hospital and Research Centre, Riyadh 11211, Saudi Arabia

**Keywords:** giant cell tumor, malignant transformation, endoprosthetic reconstruction, spindle cell sarcoma

## Abstract

**Background and Clinical Significance:** Malignant transformation of giant cell tumor of bone (GCTB) is a rare but clinically significant event, most commonly associated with radiotherapy, denosumab therapy, or recurrent disease. Secondary malignant transformation occurring in the absence of recognized risk factors is exceptionally uncommon. We report a rare case of high-grade sarcomatous transformation of proximal humeral GCTB after a prolonged latency period without prior radiotherapy, denosumab exposure, or documented recurrence; **Case Presentation:** A 27-year-old female initially presented with right shoulder pain and was diagnosed with proximal humeral GCTB. She underwent intralesional curettage and bone grafting, with histopathological confirmation of benign GCTB. Nine years later, she developed progressive shoulder pain, functional limitation, and systemic symptoms. Imaging demonstrated an aggressive lytic lesion with cortical destruction and soft-tissue extension involving the proximal humerus. Repeat curettage and histopathological evaluation revealed high-grade spindle cell sarcoma consistent with malignant transformation of GCTB. The patient received neoadjuvant chemotherapy followed by wide resection and endoprosthetic reconstruction of the proximal humerus, with additional adjuvant chemotherapy postoperatively. At two-year follow-up, she remained disease-free with excellent functional recovery and satisfactory quality of life; **Conclusions:** This case highlights the potential for delayed malignant transformation of GCTB even in the absence of established predisposing factors. Clinicians should maintain long-term vigilance in patients treated for GCTB, particularly when new pain, functional decline, or aggressive radiologic features develop years after initial treatment. Early recognition and multidisciplinary management are essential to optimize oncologic and functional outcomes.

## 1. Introduction and Clinical Significance

Giant cell tumor of bone (GCTB) is a rare, benign, and locally aggressive primary bone tumor that accounts for approximately 5% of all skeletal neoplasms and 20% of benign bone tumors [1,2]. Epidemiologic studies have demonstrated substantial geographic variation in its incidence, accounting for approximately 3–8% of primary bone tumors in Western populations compared with up to 20% in the Asian population [3]. It predominantly affects skeletally mature adults between 20 and 40 years of age [4]. Histologically, it is characterized by the presence of numerous osteoclast-like multinucleated giant cells distributed among mononuclear stromal cells, which constitute the neoplastic component [5]. Although GCTB exhibits benign biological behavior, it can recur locally, metastasize distantly, and in rare cases, undergo malignant transformation [6].

Malignant GCTB is classified as either primary, in which high-grade sarcomatous features coexist with areas of conventional GCTB at the initial diagnosis, or secondary, in which sarcoma arises at the site of a previously diagnosed benign GCTB, usually after an interval of years [7]. Secondary malignant GCTB (SMGCTB) is rare, with reported incidences ranging from 1% to 4% of all GCTBs [8]. The most frequent histologic subtypes of the malignant component are osteosarcoma, undifferentiated pleomorphic sarcoma, and high-grade spindle cell sarcoma [9].

The latency period from initial diagnosis of GCTB to the malignant transformation can vary widely, from less than 2 years to over a decade, with a mean interval of approximately 4–9 years in most series [10,11]. Established risk factors include prior radiotherapy, multiple local recurrences, and, in recent years, isolated reports have suggested a possible association between denosumab therapy and sarcomatous transformation, though causality remains unproven [12,13].

Given its rarity, prolonged latency, and the poor prognosis associated with secondary sarcomatous transformation, each case provides valuable clinical insight. Therefore, we present a rare case of a histologically confirmed benign GCTB that underwent malignant transformation to a high-grade spindle cell sarcoma nine years after the initial diagnosis. The patient voluntarily agreed to publish her relevant medical information in a scientific journal for educational purposes, and informed consent was obtained.

## 2. Case Presentation

A 27-year-old female with no significant past medical or surgical history initially presented with a three-month history of insidious onset of right shoulder pain. The pain was non-traumatic in origin, gradually progressive, and not associated with swelling, erythema, or systemic symptoms. On examination, she exhibited a limited range of motion of the right shoulder with localized tenderness over the proximal humerus and no neurovascular deficits. Plain radiographs of the right shoulder revealed a well-defined, lytic, expansile bubbly lesion involving the humeral head and proximal metaphysis, with a narrow zone of transition, intact cortical margins, and no soft-tissue component (Figure 1).

Computed tomography (CT) confirmed these features, showing a well-circumscribed osteolytic lesion with preserved cortex, no soft-tissue extension, and maintenance of the glenohumeral joint (Figure 2), findings consistent with a grade 1 Campanacci giant cell tumor of bone.

The patient subsequently underwent intralesional curettage and bone grafting, and the specimen was sent for histopathological analysis, which confirmed the diagnosis of GCTB (Figure 3).

Nine years later, at the age of 36-year-old, she presented with progressive right shoulder pain that awakens her from sleep, associated with limited range of motion, unintentional weight loss of 10 kg, anorexia, fatigue, and weakness. Plain radiographs revealed an ill-defined, permeative lytic lesion involving the metaphysis, with a wide zone of transition, cortical destruction, and an associated soft-tissue mass, features suggestive of an aggressive neoplasm (Figure 4).

Subsequently, shoulder magnetic resonance imaging (MRI) demonstrated an aggressive bony lesion in the right humeral head, with mild expansion of the proximal humerus, irregular margins, cortical destruction, and a distended joint space containing a soft-tissue component (Figure 5).

Furthermore, a positron emission tomography (PET) scan revealed a large, intensely FDG-avid lesion in the right proximal humerus with hypermetabolic soft-tissue components, as well as a few small FDG-avid right axillary and supra-/infraclavicular lymph nodes. Subsequent staging investigations, including contrast-enhanced computed tomography of the chest, abdomen, and pelvis (CT CAP), demonstrated no evidence of distant metastatic disease. Following multidisciplinary tumor board review, the FDG-avid lymph nodes were considered reactive rather than metastatic based on their imaging characteristics and clinical assessment; therefore, lymph node biopsy was not performed. The disease was considered localized, with no radiologic evidence of regional or distant metastatic disease. Subsequently, the patient underwent a repeated curettage and bone grafting, and the specimen was sent for histopathological examination, which demonstrated a high-grade pleomorphic sarcoma (Figure 6).

Following a tumor board discussion, and based on standard sarcoma management protocols, a planned neoadjuvant chemotherapy regimen consisting of three cycles of Doxorubicin (25 mg/m^2^) and Cisplatin (100 mg/m^2^) was initiated. This was followed by wide surgical resection, including the proximal epiphysis and metaphysis with circumferential soft-tissue extension and endoprosthetic reconstruction of the proximal humerus (Figure 7). Histopathological examination of the resected specimen demonstrated negative surgical margins (R0 resection), with an estimated 60–70% treatment response to neoadjuvant chemotherapy.

Postoperatively, adjuvant chemotherapy was administered with three cycles of Doxorubicin 50 mg/m^2^ and Cisplatin 70 mg/m^2^. Consequently, the patient demonstrated excellent outcomes at routine follow-up at 6 months, 9 months, and 2 years, achieving a score of 1 on the European Organisation for Research and Treatment of Cancer Quality of Life Questionnaire, Core 30 (EORTC QLQ-C30), indicating an excellent functional and quality-of-life measure.

## 3. Discussion

Malignant transformation of GCTB is an uncommon but clinically significant event, with secondary malignant GCTBs representing a minority of such transformations [8]. In our case, the nine-year interval between the initial benign GCTB presentation and the subsequent high-grade spindle cell sarcoma transformation highlights the potential for late malignant changes and emphasizes the need for long-term surveillance.

GCTB is a locally aggressive tumor with unpredictable behavior and a high rate of local recurrences [14]. It typically arises in the meta-epiphyseal region of long bones, especially the distal femur, proximal tibia, and distal radius, although virtually any skeletal location may be affected [15]. The tumor predominantly occurs in skeletally mature individuals, with peak incidence between the second and fourth decades of life, with a slight female predominance [16]. Patients with GCTB typically present with localized pain, swelling, functional limitation, and, in rare cases, a pathological fracture may be the initial presentation due to substantial cortical destruction [14].

Diagnosing GCTB is based on a combination of clinical, radiological, and histopathological findings. Plain radiographs typically reveal an eccentric, lytic, expansile lesion with well-defined and non-sclerotic margins. MRI assists in defining intramedullary and soft-tissue extension, while CT offers a detailed assessment of cortical integrity [17]. Histopathology remains the gold standard, showing mononuclear stromal cells interspersed with multinucleated osteoclast-like giant cells [5]. The Campanacci grading system is the most widely used classification for GCTB [15], categorizing lesions based on radiographic appearance: Grade I lesions are well-marginated with an intact cortex, Grade II lesions are relatively well defined with cortical thinning or expansion but without cortical breakthrough, while Grade III lesions demonstrate poorly defined margins with cortical destruction and extension into the surrounding soft tissues.

The mainstay of treatment for GCTB is surgical excision, with options including intralesional curettage with bone grafting or en bloc resection. Intralesional curettage with high-speed burring preserves joint function; however, it carries a substantially higher risk of local recurrence, reaching approximately 49% [18]. Conversely, en bloc resection can achieve nearly 100% local control but is commonly associated with long-term functional impairment due to extensive bone destruction [19].

Malignant transformation of GCTB, while rare, may occur de novo (primary malignant GCTB) or as a secondary event following prior treatment of a benign lesion (secondary malignant GCTB). Reported risk factors include previous radiotherapy, which is historically the most recognized cause, repeated local recurrence, and, more recently, concerns about prolonged denosumab therapy [20,21]. Denosumab, a RANKL inhibitor, has shown efficacy in unresectable or complex GCTB cases; however, cases of sarcomatous transformation during or after denosumab treatment have been reported, raising questions about optimal duration and monitoring [22,23,24]. Importantly, secondary malignant GCTB can develop many years after initial treatment without any identifiable predisposing factors, as illustrated in our case.

The present case is particularly noteworthy because malignant transformation occurred in the absence of the most commonly reported risk factors, including prior radiotherapy, denosumab exposure, and documented local recurrence. Although SGCTB is itself rare, most reported cases have been associated with one or more of these risk factors. In contrast, our patient developed a high-grade pleomorphic sarcoma nine years after treatment of a histologically confirmed benign GCTB without evidence of recurrence or exposure to recognized oncologic risk factors. As summarized in Table 1, only a limited number of similar cases have been reported in the literature, highlighting the exceptional rarity of this clinical presentation and emphasizing the importance of maintaining long-term surveillance even in patients considered to be at relatively low risk for malignant transformation.

Beyond clinical management, growing preclinical and translational research efforts are improving our understanding of GCTB biology and the mechanisms underlying malignant transformation. Recent studies have highlighted the critical role of the tumor microenvironment, including interactions between neoplastic stromal cells, osteoclast-like giant cells, immune cells, and the bone matrix, in driving tumor progression and treatment response [27,28]. Additionally, advanced experimental platforms, such as patient-derived primary cultures, three-dimensional (3D) scaffold-based models, and tissue-engineered tumor systems, have enabled more accurate recapitulation of the bone tumor microenvironment compared with conventional two-dimensional cultures [27,29]. These models provide valuable tools for investigating tumor–stromal interactions, mechanisms of treatment resistance, and potential therapeutic targets in GCTB and related bone sarcomas. Furthermore, translational studies integrating molecular profiling with 3D culture systems may facilitate the identification of biomarkers associated with aggressive behavior and malignant transformation, ultimately supporting the development of more personalized treatment strategies for patients with GCTB [28].

Despite its rarity, malignant transformation of GCTB carries a worse prognosis compared with conventional GCTB. Reported survival outcomes remain poor, with Liu et al. [10] describing 5-year survival rates of approximately 40% in secondary malignant cases. Similarly, Bertoni et al. [11] observed that patients with secondary malignant GCTB, particularly those following radiotherapy, generally experienced poor outcomes. These findings collectively highlight the aggressive nature of secondary malignant GCTB and emphasize the necessity for early recognition and vigilant long-term follow-up.

This report has several limitations. As a single-case report, the findings may not be generalizable to all patients with secondary malignant GCTB. In addition, although the patient remained disease-free with satisfactory functional outcomes at two years of follow-up, the relatively short oncologic follow-up limits assessment of long-term local control, metastatic progression, and overall survival. Given the potential for late recurrence and disease progression in malignant GCTB, continued long-term surveillance remains essential.

## 4. Conclusions

Secondary malignant transformation of a giant cell tumor of bone, although rare, represents a serious and potentially life-threatening event. Our case highlights that such transformation can occur nearly a decade after initial treatment of a benign GCTB, emphasizing the importance of long-term surveillance for early detection. Accurate diagnosis, timely surgical intervention, and appropriate adjuvant therapy are essential to optimize functional outcomes and improve survival.

## Figures and Tables

**Figure 1:**
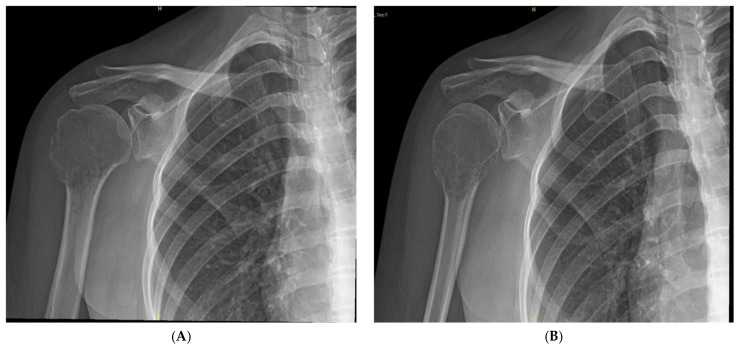
(**A**) Anteroposterior radiograph of the right shoulder showing a well-defined expansile osteolytic lesion in the epiphyseal–metaphyseal region of the proximal humerus with cortical thinning. (**B**) Lateral radiograph demonstrating the lesion without cortical breach or soft-tissue extension, consistent with giant cell tumor of bone.

**Figure 2:**
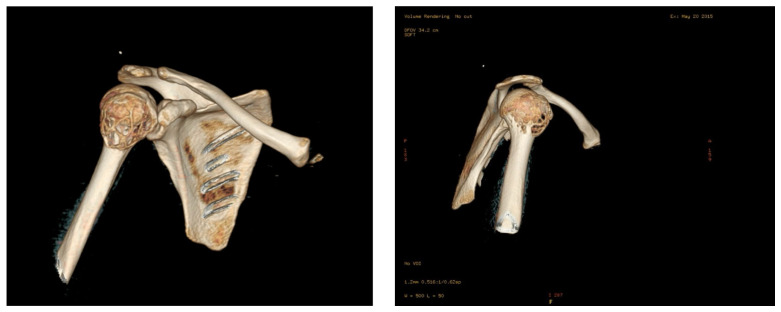
CT scan of the proximal humerus demonstrating a well-circumscribed osteolytic lesion with preserved cortex and no soft-tissue extension.

**Figure 3:**
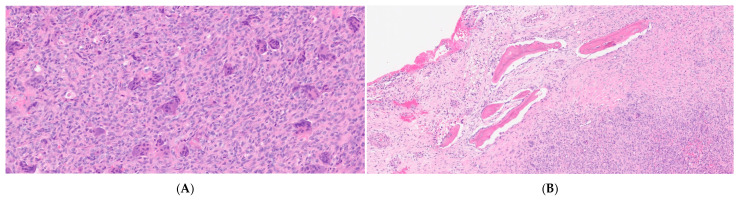
Histopathological features of the initial benign giant cell tumor of bone (GCTB). (**A**) Classical histomorphology of giant cell tumor of bone showing a proliferation of relatively uniform mononuclear stromal cells, admixed with numerous evenly distributed osteoclast-type multinucleated giant cells. The stromal cells exhibit bland cytologic features without significant nuclear pleomorphism. No tumor necrosis, atypical mitotic figures, or significant cytologic atypia are identified (H&E stain, original magnification ×20). (**B**) Peripheral reactive bone formation is present at the edge of the giant cell tumor, characterized by well-formed trabeculae lined by prominent osteoblastic rimming. No cytologic atypia, malignant osteoid production, or other features suggestive of malignant transformation are identified (H&E stain, original magnification ×20).

**Figure 4:**
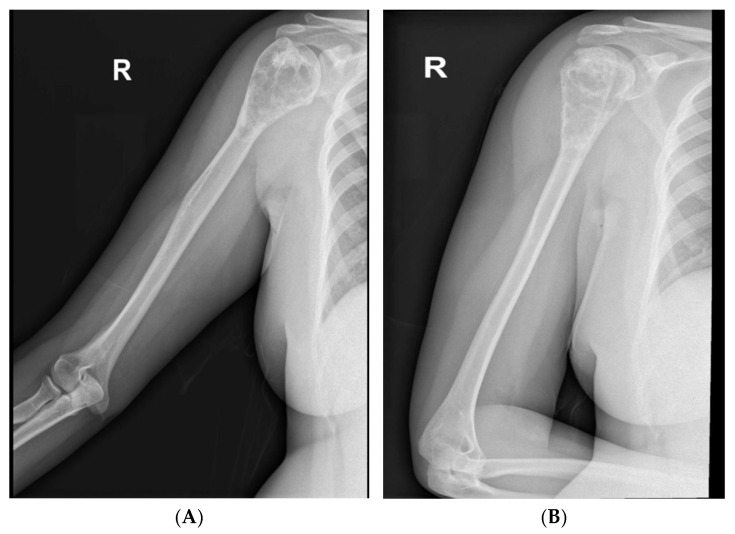
(**A**) Anteroposterior radiograph of the right shoulder demonstrating an ill-defined permeative osteolytic lesion involving the proximal humerus with cortical destruction. (**B**) Lateral radiograph showing associated soft-tissue extension, consistent with an aggressive lesion.

**Figure 5:**
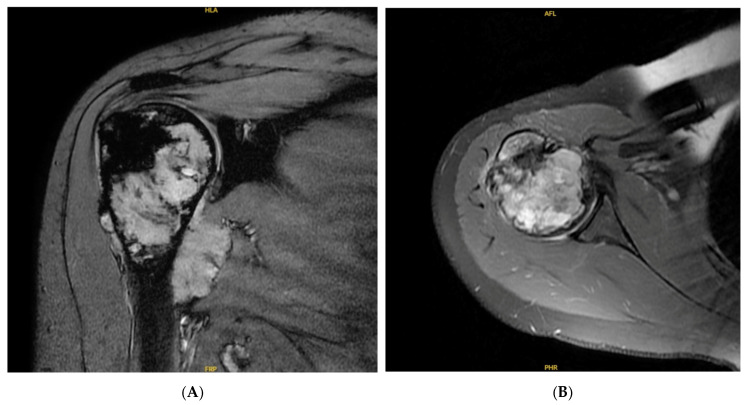
(**A**) Coronal MRI of the right shoulder demonstrating an aggressive lesion involving the proximal humerus with cortical destruction. (**B**) Axial MRI showing associated soft-tissue extension and joint involvement, suspicious for malignant transformation.

**Figure 6:**
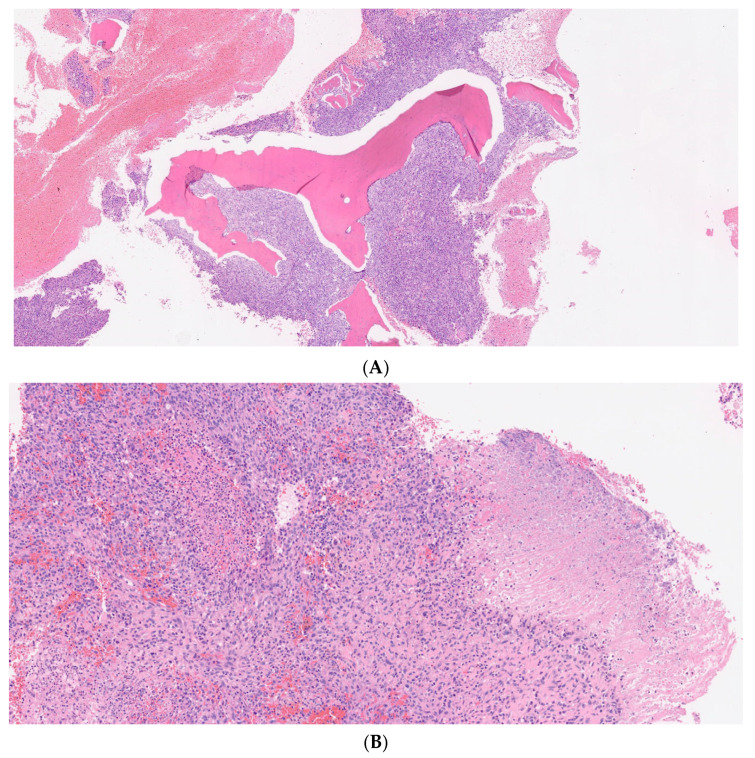

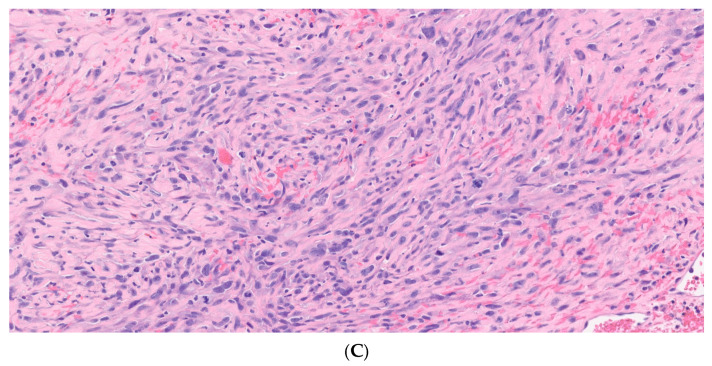
Histopathological features of malignant transformation. (**A**) Malignant transformation characterized by an infiltrative high-grade sarcomatous neoplasm permeating and entrapping pre-existing host bone trabeculae. Destructive invasion of native bone is a key histologic feature supporting malignancy and is not typically encountered in conventional giant cell tumor of bone or other benign/intermediate giant cell-rich bone lesions (H&E stain, original magnification ×4). (**B**) Intermediate-power photomicrograph demonstrating a pleomorphic high-grade sarcoma composed of markedly atypical malignant cells. A focus of tumor necrosis is present on the right side of the image (H&E stain, original magnification ×20). (**C**) High-power view showing a pleomorphic high-grade sarcoma composed of atypical spindle to polygonal cells with marked nuclear atypia, brisk mitotic activity, and focal eosinophilic matrix suspicious for osteoid formation. Only rare osteoclast-type giant cells are present, with no definite residual conventional giant cell tumor component identified (H&E stain, original magnification ×40).

**Figure 7:**
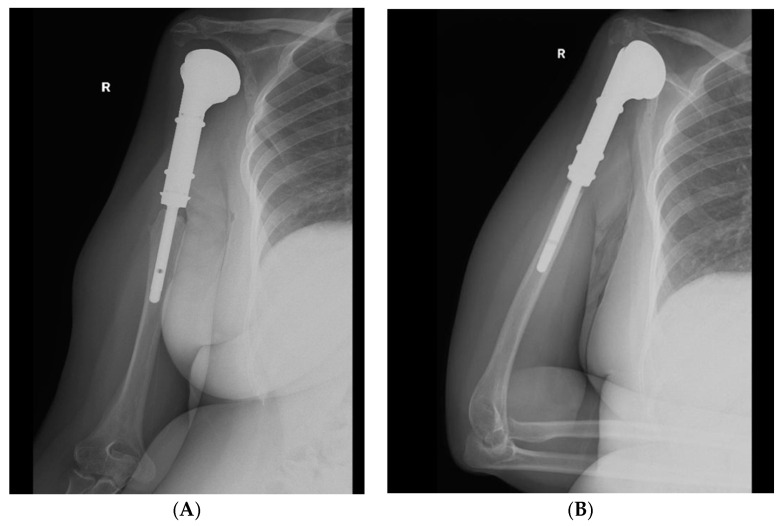
(**A**) Anteroposterior radiograph of the right shoulder demonstrating proximal humeral resection with endoprosthetic reconstruction. (**B**) Lateral radiograph showing appropriate alignment and positioning of the proximal humeral endoprosthesis.

**Table 1:** Reported Cases of Secondary Malignant GCTB.

Study	Age	Sex	Primary Site	Risk Factors	Time to MT	MT Histology
**Alshaygy et al., (current study)**	27	Female	Right proximal hummers	Recurrence: No RT: No Denosumab exposure: No	9 years	High-grade pleomorphic sarcoma
**Miller et al. [25]**	29	Male	left proximal tibia	Recurrence: Yes RT: No Denosumab exposure: No	4 years	Undifferentiated Pleomorphic Sarcoma
**Broehm et al. [23]**	46	Male	Right pelvis	Recurrence: Yes RT: No Denosumab exposure: Yes	13 years	Osteosarcoma
**Broehm et al. [23]**	49	Male	Left femur	Recurrence: Yes RT: No Denosumab exposure: Yes	7 years	Osteosarcoma
**Takesako et al. [26]**	75	Male	Left distal femur	Recurrence: No RT: No Denosumab exposure: No	40 years	Undifferentiated Pleomorphic Sarcoma
**Tsukamoto et al. [24]**	29	Female	Left ischium	Recurrence: Yes RT: No Denosumab exposure: Yes	10 years	Osteosarcoma

MT, malignant transformation; RT, radiotherapy.

## Data Availability

The original data presented in the study are included in the article. Further inquiries can be directed to the corresponding authors.

## Abbreviations

The following abbreviations are used in this manuscript:

GCTB	Giant Cell Tumor of Bone
SMGCTB	Secondary Malignant Giant Cell Tumor of Bone
CT	Computed Tomography
MRI	Magnetic Resonance Imaging
PET	Positron Emission Tomography
FDG	Fluorodeoxyglucose
RANKL	Receptor Activator of Nuclear Factor Kappa-B Ligand
EORTC QLQ-C30	European Organisation for Research and Treatment of Cancer Quality of Life Questionnaire Core 30
